# From Sensor Data to Animal Behaviour: An Oystercatcher Example

**DOI:** 10.1371/journal.pone.0037997

**Published:** 2012-05-31

**Authors:** Judy Shamoun-Baranes, Roeland Bom, E. Emiel van Loon, Bruno J. Ens, Kees Oosterbeek, Willem Bouten

**Affiliations:** 1 Computational Geo-Ecology, Institute for Biodiversity and Ecosystem Dynamics, University of Amsterdam, Amsterdam, The Netherlands; 2 Department of Marine Ecology and Evolution, Royal Netherlands Institute for Sea Research (NIOZ), AB Den Burg, Texel, The Netherlands; 3 SOVON Dutch Centre for Field Ornithology, Coastal Ecology Team, AB Den Burg, Texel, The Netherlands; Cajal Institute - Consejo Superior de Investigaciones Científicas, Spain

## Abstract

Animal-borne sensors enable researchers to remotely track animals, their physiological state and body movements. Accelerometers, for example, have been used in several studies to measure body movement, posture, and energy expenditure, although predominantly in marine animals. In many studies, behaviour is often inferred from expert interpretation of sensor data and not validated with direct observations of the animal. The aim of this study was to derive models that could be used to classify oystercatcher (*Haematopus ostralegus*) behaviour based on sensor data. We measured the location, speed, and tri-axial acceleration of three oystercatchers using a flexible GPS tracking system and conducted simultaneous visual observations of the behaviour of these birds in their natural environment. We then used these data to develop three supervised classification trees of behaviour and finally applied one of the models to calculate time-activity budgets. The model based on accelerometer data developed to classify three behaviours (fly, terrestrial locomotion, and no movement) was much more accurate (cross-validation error = 0.14) than the model based on GPS-speed alone (cross-validation error = 0.35). The most parsimonious acceleration model designed to classify eight behaviours could distinguish five: fly, forage, body care, stand, and sit (cross-validation error = 0.28); other behaviours that were observed, such as aggression or handling of prey, could not be distinguished. Model limitations and potential improvements are discussed. The workflow design presented in this study can facilitate model development, be adapted to a wide range of species, and together with the appropriate measurements, can foster the study of behaviour and habitat use of free living animals throughout their annual routine.

## Introduction

Understanding how animals interact with their environment is one of the fundamental aims of animal ecology. In order to acquire this knowledge we need information which can be used to quantify what animals are doing, when, where, how and for how long. For a broad spectrum of ecological research, from theoretical to applied, quantitative time budget information at the individual level is important [1]–[3]. A quantitative approach can provide essential information for species and habitat conservation [4]–[5], understanding ecosystem dynamics [2], [6], understanding and mitigating the spread of animal borne diseases [7]–[8], animal adaptation to climate and landuse change [4], [9], spread of introduced and invasive species [3] and the development of environmental policy [10]. For example, when addressing the direct and indirect impact of fisheries on seabirds (see question 26 [10]), we would like to know where, when and how a species forages [2], [11]–[12].

Our ability to visually observe the behaviour of free-ranging animals is generally quite restricted in space and time. In recent decades, technological advances have enabled researchers to track animals during local and migratory movements, in the air, on land and in the sea [13]–[17]. Similarly, bio-logging features such as body acceleration, heart rate, stomach temperature, diving depth enable remote monitoring of an animal's physiological state and its activity in 3 dimensional space and in time [18]–[20]. The data collected by these sensors can then be used to infer what an animal is doing. For example, speed measured directly using GPS (global positioning system) or derived from consecutive tracking locations has been used to infer behaviour, to distinguish between travelling and resting during migration [21]–[23], and during foraging trips [24]–[25]. Yet, instantaneous speed measured with a GPS is probably too inaccurate for distinguishing small differences in locomotion, especially at low speeds [26]. Accelerometers are a promising sensor for studying animal behaviour remotely since accelerometers can measure the posture and body movements ([27] and references therein) as well as estimate the speed and energy expenditure [3], [28]–[30] of the animal to which it is attached. In the last decade, dynamic and static body acceleration have been used to study a diverse range of behaviours including diving [31]–[33], swimming and flight strategy [34]–[37], feeding and breathing [38] and mating behaviour [39]. Behavioural studies utilizing accelerometer data have focused primarily on marine animals [40] and very few studies have focused on terrestrial locomotion in wild animals [3], [41]–[43].

Quantifying behaviour from bio-logging data requires an intermediate step to translate the measured sensor data into specific behaviours. Three general approaches to achieve this translation are: (1) non-automated interpretation of sensor data by an expert, with [2], [44]–[45]) or without [35], [41] field observations of the animal's behaviour; (2) automated segmentation or clustering of sensor data without field observations of animal behaviour, sometimes followed by labelling of the identified segments by an expert [46]; (3) automated classification of sensor data in combination with observations of the animal's behaviour [3], [38], [47]. For brevity we will call the first method *expert interpretation*, the second *clustering*, and the third method *classification*. In most studies of wild animals, behaviour has been inferred by expert interpretation; however, the inferred behaviour cannot be validated via this method. In cases where behavioural observations are not available, only the methods of expert interpretation and clustering can be applied to sensor data. The essential difference between the two methods is that for expert interpretation the behavioural classes have to be specified prior to the classification task, whereas in clustering the specification (and meaning) of behavioural classes follow from the clustering results. Hence, expert interpretation is deductive whereas the clustering is inductive in nature. Due to the lack of behavioural observations which match the sensor data, the uncertainty of the results cannot be assessed for either of these methods. In contrast, the classification method can provide information about the uncertainty of the classification result. Knowledge of classification uncertainty can be used to answer various kinds of inferential questions such as whether a given model (using sensor data) is able to predict behaviour better than a null-model or whether a given number of behavioural classes can be distinguished.

The primary aim of this study was to derive, evaluate and compare models to classify measurements from sensors attached to individual shorebirds, the oystercatcher (*Haematopus ostralegus*), into pre-defined behaviours; one model would only be based on speed measured by GPS and other models would include accelerometer data. The use of accelerometer data to remotely determine the behaviour of terrestrial wild animals is still quite new. We expected that classification models based on GPS-speed alone would improve if accelerometer data would be included. We describe a methodological workflow that we used in this study to develop and apply classification models of animal behaviour. The aim of such a workflow is to provide researchers with a clear outline of the diverse processing and analysis steps needed to quantify behaviour based on sensor data; it can be applied to other studies, streamline analysis of new data and the reanalysis of existing data. By using sensor data to quantify behaviour in combination with location data, time-activity budgets of animals can be quantified at the individual level and in relation to their environment [19]. To show the added value of incorporating behavioural information with location data we calculated the time-activity budget of an individual bird for areas and times of day that are normally difficult to visually observe in the field. More specifically, we wanted to determine if an individual spends its time differently during the day compared to during the night and how does it spend its time when outside the territory.

## Methods

### Study species and study area

**Figure 1:**
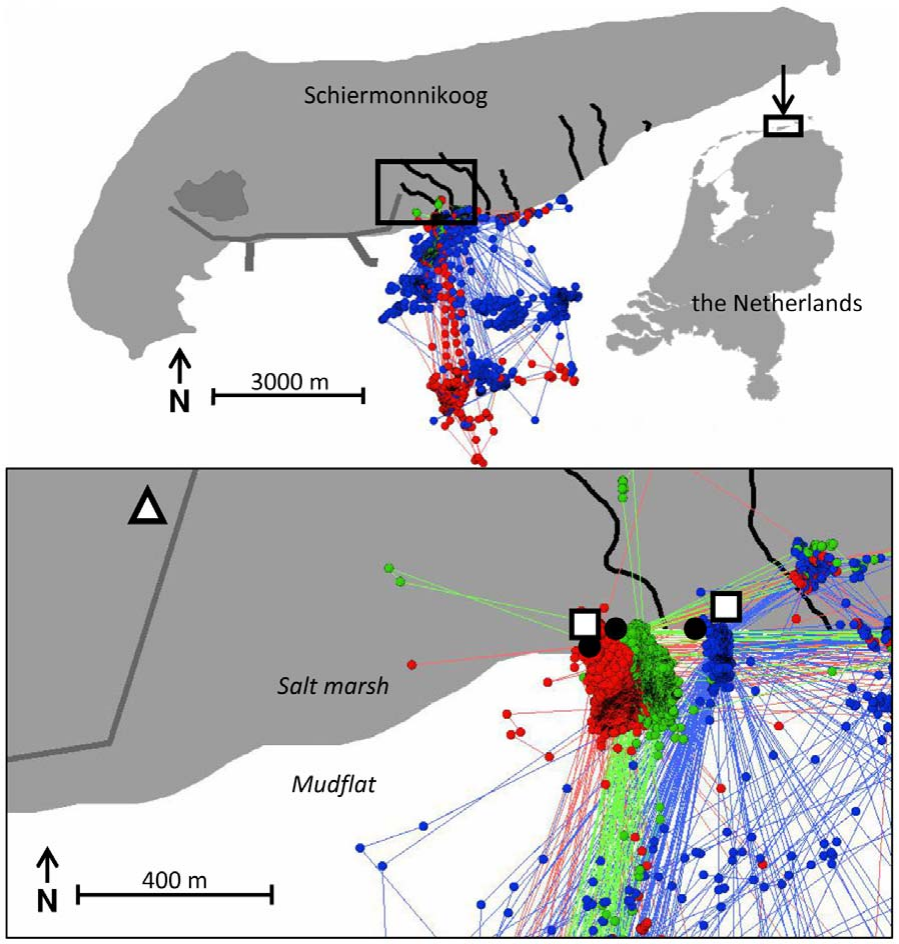
The study area on the island of Schiermonnikoog, the Netherlands (53.29°N, 06.10°E) at different spatial scales. The points represent GPS fixes of three oystercatchers (green – tag 166, red – tag 167, blue – tag 169; Table S1) from 1 July 2009 to 31 July 2009, with consecutive points connected by lines. The black circles are the nests of these birds. The locations of the observation towers are indicated by a square and the base station by a triangle. Black lines represents creeks, dark grey lines represent urban infrastructure.

The oystercatcher is a long lived, monogamous wader that feeds on intertidal prey, such as hard-shelled bivalves they can open with their strong bill and large marine worms. They breed predominantly in coastal habitats, although inland breeding increased during the second half of the previous century [48]. On the Dutch Wadden Island Schiermonnikoog (53.26°N, 06.10°W, Figure 1) a population of oystercatchers has been studied and individuals have been colour ringed since the 1983 (e.g. [49]–[51]). In this population, colour ringed individuals can be easily identified and a range of behaviours can be visually observed in the field from two observation towers (Figure 1).

### Methodological workflow

**Figure 2:**
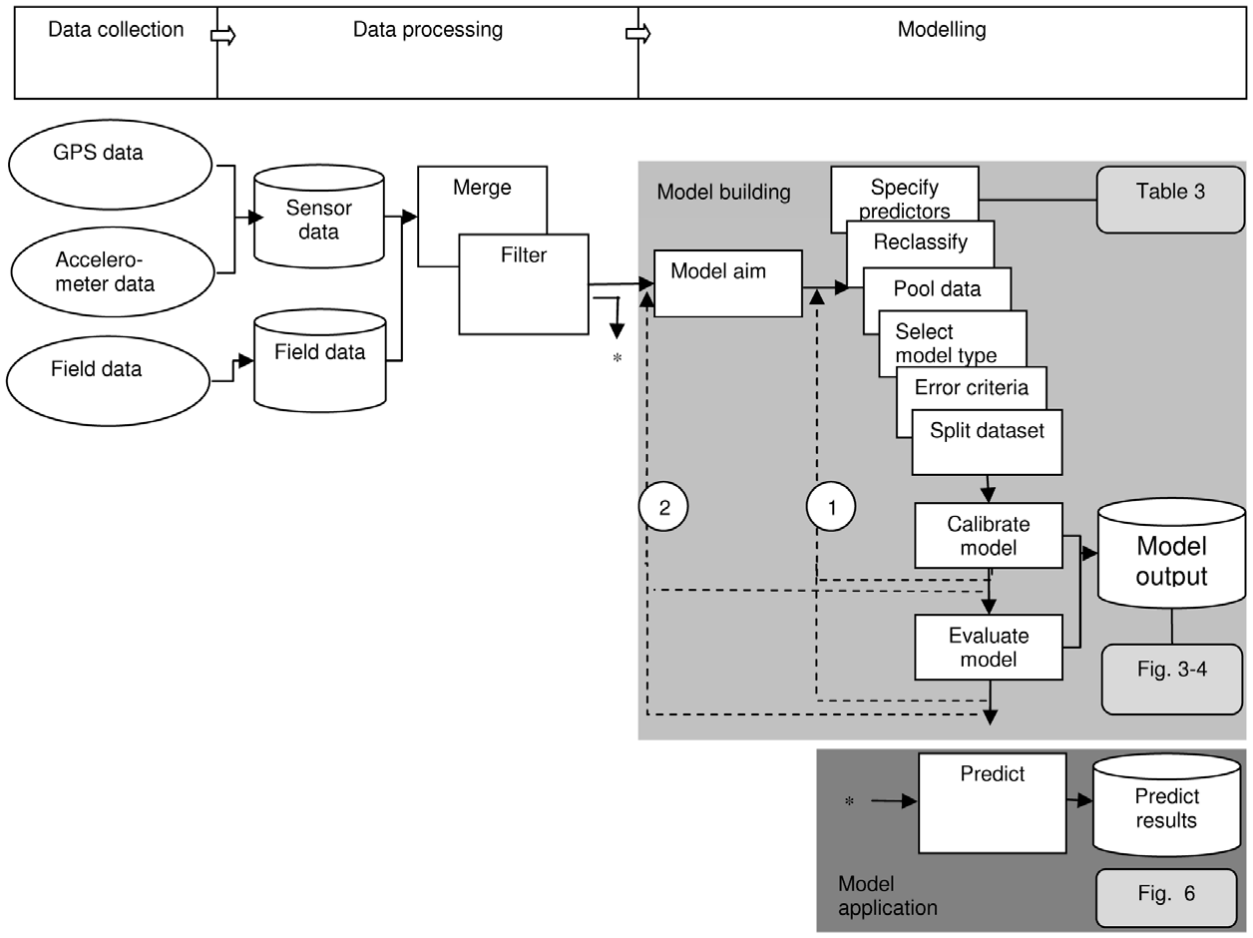
A schematic workflow of the different methodological steps conducted in this study. The workflow is broken down into three main categories of activity shown on the upper bar: Data collection, Data processing and Modelling. The objects in the grey rectangle indicate the aspects involved in building classification models and the objects in the dark grey rectangle indicate application of the classification models for diverse analyses such as calculating time budgets. Ovals indicate data in various formats (files from data loggers, written field forms, etc). Cylinders indicate information that is stored in a database. White rectangles indicate (computational) activities and decisions. Solid arrows present the workflow to move from field data to the establishment and application of a model. Dashed arrows present feed-back loops where a certain part of the workflow is repeated in response to progressive insights (only the most important feed-back loops are shown). Feed-back loops are present from a point after model calibration as well as a point after model evaluation back to the beginning of the modelling sequence (2) or later in the modelling sequence (1). These steps are generalized so that they can be applied to other studies, for example visual observations may be replaced by video observations or expert interpretation of sensor data.

In the following sections we briefly describe how each of the following research steps was applied in the current study: data collection, data processing, modelling and model application. A more detailed description is provided in Text S1. The steps are also shown in a schematic workflow diagram (Figure 2) and present a general methodological approach that can be applied to any study where measurements from sensors attached to animals will be used in combination with observations of behaviour to derive and apply a classification model of animal behaviour.

### Data collection

In this study, we used the recently developed UvA Bird Tracking System (UvA-BiTS, University of Amsterdam Bird Tracking System) which has been used to study several resident and migratory bird species (e.g. [24]). The tracking device is solar-powered and weighs 13.5 g, and includes a tri-axial accelerometer and a GPS receiver which measures geographic position, altitude above mean sea level, time and instantaneous speed. The tri-axial accelerometer measurements were converted to acceleration in g (1 g = 9.8 m s^−2^) with respect to the earth's gravitational field in three directions: surge (X), sway (Y) and heave (Z).

During the breeding season of 2009 (May-July 2009), oystercatchers were observed for several weeks and three colour ringed birds breeding in high quality territories adjacent to the mudflats were selected for our tracking study and trapped towards the end of the breeding season (Table S1). Observations in the breeding area prior to trapping were described in more detail in previous studies [50]–[52]. The birds were caught on their nest with a walk-in trap. After the birds were weighed and morphological measurements were taken, a tracking device was fitted on their back using a Teflon ribbon harness (weight ∼2 g). The harness was attached to the bird using a figure eight configuration. The straps were connected around the neck and the wings to one weak point at the sternum. The weak point was made out of cotton thread, which is expected to deteriorate in two to three years. The harness and tracking device weighed less than 3% of the mean body mass of the birds (Table S1). Birds were released within 60 minutes of capture.

A GPS fix was taken every 10 minutes from 30 June 2009 through 20 July 2009 and every 30 minutes from 21–31 July. Directly following each GPS fix, acceleration was measured with a frequency of 20 Hz for 3 seconds. From 30 June through 14 July 2009 each bird was observed daily for 30 minutes with a telescope (20–60×, Zeiss Diascope 85 T*FL) positioned in one of the observation towers (Figure 1). During visual observations, the tracking device was set to take a GPS fix at 10 s intervals followed by 3 seconds of acceleration measurements. When a bird started a new behaviour, it was reported by the observer and recorded by a field assistant in a PSION handheld computer (Workabout Pro) with Observer XT software (www.noldus.com). To accurately link the visual observations with the GPS and accelerometer measurements, the handheld computer was synchronized to GPS time using a handheld GPS. The recording procedure was first practised extensively on non tagged birds. The main behaviours defined by Kersten [53] were extended with sub-behaviours observed in the field (Table 1); the classes to express the behaviours as well as the sub-behaviours are both exhaustive and exclusive.

**Table 1:** Different behaviours and sub-behaviours visually observed during the study and linked to GPS and accelerometer measurements.

Behaviour	Sub behaviour	Description	3-class model behaviours	*n*
Aggression	Bobbing	Bird is standing and moves its body up and down	No locomotion	4
	Chasing	Bird is chasing conspecifics	Terrestrial locomotion	3
	Stand Solitary piping	Bird is calling loudly while standing, conspecifics are nearby	No locomotion	18
	Piping ceremony	Bird is calling loudly together with other birds, while walking	Terrestrial locomotion	12
	Walk Solitary piping	Bird is calling loudly while walking, conspecifics are nearby	Terrestrial locomotion	12
Body care	Preen	Bird is preening its feathers	No locomotion	82
	Wash	Bird is bathing	No locomotion	3
Fly	normal flight	Bird is flying	Fly	13
Forage	By sight	Bird is searching for prey by sight while walking	Terrestrial locomotion	249
	By touch	Bird is searching for prey by touch while walking	Terrestrial locomotion	5
Handle	Handling at surface	Bird is handling the prey at the surface	No locomotion	15
	Handling in situ	Bird is handling the prey beneath the surface	No locomotion	29
	Walking with prey	Bird is walking with the prey	Terrestrial locomotion	7
Sit		Bird is sitting	No locomotion	100
Stand		Bird is standing	No locomotion	125
Walk		Bird is walking	Terrestrial locomotion	25

The column ‘3-class model behaviours’ shows the behavioural classes reclassified a priori and used to calibrate the 3-class models (S3 and SA3). The behavioural classes in the first column were used as the predicted variable in the 8-class model (SA8). The number of visual observations (*n*) is provided per behaviour.

### Data processing

In this study data processing (Figure 2) included data storage, merging datasets and filtering data. All the GPS and accelerometer data that were collected during the study period were stored in a dedicated postgreSQL database (http://www.uva-bits.nl/virtual-lab/) and the visual observations were stored in a separate data base. This enables researchers to systematically explore or re-use (parts of) the data sets if needed. The GPS and accelerometer data were labelled with the visually observed behaviours, while accounting for a maximum of 10 s recording delay on the handheld computer (‘merge’ in Figure 2), see Text S1 for more details. Next, all data were checked for anomalies (‘filter’ in Figure 2). For example, data that could not be unambiguously linked to a behavioural observation were removed from further analysis.

### Modelling

Note that the decisions made during the model building phase (grey ‘model building’ rectangle in Figure 2) regarding the data, model design and analysis steps are dependent on each other and, in general, can also be dealt with in a different order than chosen here. One of the first steps in our analysis was defining the model aim (‘model aim’ in Figure 2) which was to accurately predict behaviour, whereby all behavioural classes were considered equally important. In this study, we report three modelling cycles, each leading to a different model. We first start a simple model with three behavioural classes and incrementally working towards more detailed models. The aim of the first model (‘model S3’, speed model of three behaviour classes) was to predict three behaviour classes. The predictor variable specified for this model (‘specify predictors’ in Figure 2) was GPS speed. The original behaviour classes were grouped (‘reclassify’ in Figure 2) into three behaviour classes (Table 1, column 4). With the aim of predicting three behaviour classes, we then went through feedback loop 1 to develop the second model (‘model SA3’, speed-acceleration model of three behaviour classes) using predictor variables based on accelerometer data, described in more detail below, as well as GPS speed.

While speed was provided by the GPS sensor, the acceleration measurements had to be processed to calculate meaningful predictor variables. We derived 15 predictor variables from the tri-axial acceleration segments (measurement frequency of 20 Hz for 3 seconds). All predictor variables used in this study are listed in Table 2 (see Text S1 for more detailed information on how the accelerometer data were processed). All the predictors, except for the mean dynamic body acceleration in single dimensions (odbaX, odbaY and odbaZ), have been used in other studies [27], [35], [44], [47] and are described in [47]. The overall dynamic body acceleration (odba) was calculated as the sum of odbaX, odbaY and odbaZ and has been used in other studies as a single measure of body movement and a potential proxy for energy expenditure, see [30] for a detailed explanation.

**Table 2:** Predictive parameters used in this study, derived from the GPS (speed) and the accelerometer sensors.

predictor	direction	label	explanation
GPS speed (m s^−1^)	-	speed	3D speed
body pitch (°)	surge	pitchX	angle of the body along the surge axis
	heave	pitchZ	angle of the body along the heave axis
body roll (°)	sway	rollY	angle of the body along the sway axis
maximum dynamic body acceleration (g)	surge	mdbaX	maximum dynamic body acceleration along the surge axis
	sway	mdbaY	maximum dynamic body acceleration along the sway axis
	heave	mdbaZ	maximum dynamic body acceleration along the heave axis
overall dynamic body acceleration (g)	surge	odbaX	Mean dynamic body acceleration along the surge axis
	sway	odbaY	Mean dynamic body acceleration along the sway axis
	heave	odbaZ	Mean dynamic body acceleration along the heave axis
	-	odba	overall dynamic body acceleration (odbaX+odbaY+odbaZ)
dominant power spectrum (g^2^Hz^−1^)	surge	dpsX	maximum power spectral density (psd) of dynamic acceleration along the surge axis
	sway	dpsY	maximum psd along the sway axis
	heave	dpsZ	maximum psd along the heave axis
frequency at the dominant power spectrum (Hz)	surge	fdpsX	frequency at the maximum psd along the surge axis
	sway	fdpsY	frequency at the maximum psd along the sway axis
	heave	fdpsZ	frequency at the maximum psd along the heave axis

The dominant power spectrum measures the relative amount of kinetic energy that is spent at the dominant periodicity in a signal (see Text S1 for more details).The integration interval of the measurement for the accelerometer sensor is 3 seconds with 20 Hz. The direction in which each variable is defined is given in Cartesian coordinates relative to the ground surface: surge represents the x-axis, sway the y-axis and heave the z-axis. The measurement units (SI) are provided in parentheses.

In the third modelling cycle (‘model SA8’, speed-acceleration model of eight behaviour classes) we went through feedback loop 2; the model aim is to classify the eight main behavioural classes (Table 1, column 1) using all available predictors. In order to ensure a sufficient sample size per behaviour (Table 1, column 5) to train the models, the observations of the three individuals were pooled (‘pool data’ in Figure 2), treating each individual observation and each individual equally. Even after pooling the data, the sample size was very small for several of the sub-behaviours.

We selected classification trees (‘select model type’ in Figure 2) as our modelling approach [54]–[55], using the implementation in the rpart R package [56]–[57]. Overall cross-validation error was used as a single criterion to measure the degree of success (‘error criteria’ in Figure 2). We did not split the data into sub-sets for model calibration and evaluation because the dataset was already limited in size (with only a few observations for some behavioural classes) and also because data splitting is an integral part of the model calibration procedure for regression trees as described below.

Classification trees were derived (‘calibrate model’ in Figure 2) by initially growing a maximum (over-fitted) tree, which was subsequently pruned to an optimal size. To determine the optimal tree size, we applied the ‘one standard deviation rule’: select the smallest tree whose cross validation error is less than the minimum cross validation error +1 standard deviation [54]. Model performance was evaluated (‘evaluate model’ in Figure 2) by 10-fold cross-validation in which the dataset is split into 10 partitions, 9 of which are used to calibrate the model and 1 is used to evaluate the model; the calibration and evaluation is then repeated 10 times using a new data partition [58]. Once a model could not accurately predict behaviour, the modelling cycle would not be repeated to predict behaviour in more detail.

### Model application

To exemplify model application we calculated time budgets per individual, including areas and times of day that are difficult to visually observe in the field. We applied model SA8 to the sensor data collected in July 2009, a period for which visual observations were not collected, to classify each data point into discrete behaviours associated to the geographic position provided by the GPS (‘predict’ in Figure 2). We then used the predicted behaviours to calculate the time budget during the day and at night for three different habitats (territory, mudflats and salt marsh). For more details see Text S1.

The percentage of time devoted to each of the classified behaviours was calculated by dividing the number of observations per behaviour by the total number of observations during the day or during the night. We associated each GPS fix to one of the following habitats: territory, mudflats and salt marsh using the geographic database of global administrative areas (GADM, http://www.gadm.org). A bird was considered to be in its territory when it was within 150 m from its nest. This distance was chosen after visually inspecting the locations of each individual in relation to their nest. All terrestrial areas outside the territory, which are predominantly salt marsh in the study area, were labelled salt marsh. The inter-tidal areas were labelled mudflats. Day was defined as the hours between sunrise and sunset at Schiermonnikoog (53.47 N, 6.23 E).

### Software implementation of the various analysis steps

The data processing steps, the definition of prediction variables and subsequent modelling were conducted using the R language for statistical computing [57]. We provide a modelling package with the scripts developed for data analysis (model building and model application) and the data presented in this study (Dataset S1).

## Results

### Behavioural measurements

Table S1 provides an overview of the number of GPS and acceleration segments (60 measurements per segment) collected for each bird. During visual observations 16 behaviours were observed and 702 GPS fixes and acceleration segments could be linked to the visual observations (Table 1). Forage by sight was the most frequently observed behaviour. The mean value of each predictor variable is provided per observed behaviour in Tables S2A–C. Mean speeds did not differ significantly between behaviours within the no locomotion and terrestrial locomotion behaviours (*P*>0.05, Tukey HSD test). This justified the reclassification of behaviours into three categories, fly, terrestrial locomotion and no locomotion, before fitting the speed model (Table 1, Column 4).

### 3-class speed model

**Figure 3:**
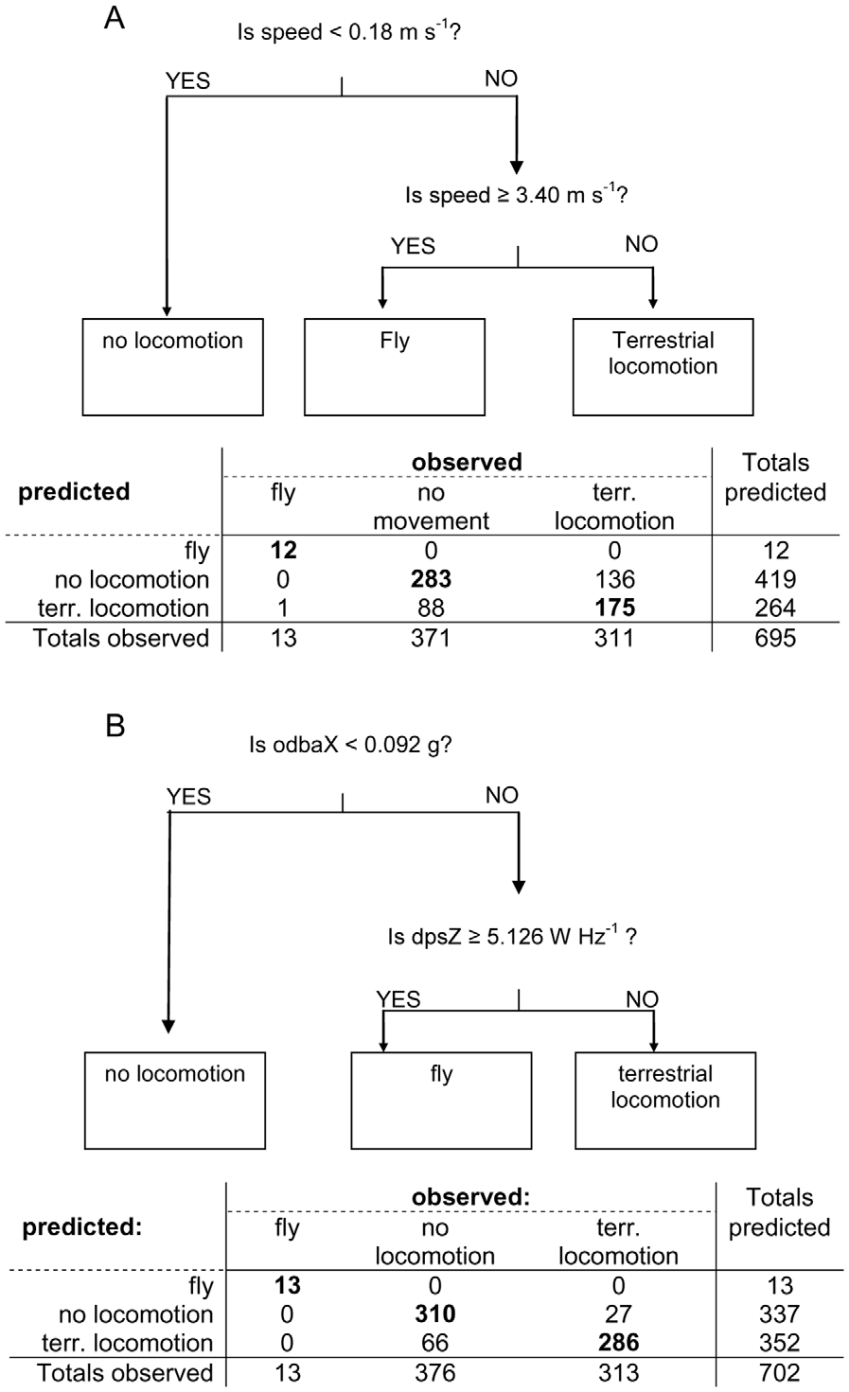
Decision tree and confusion matrix for models S3 and SA3. For model S3 (A) and model SA3 (B), the number of observations correctly classified per behaviour is shown in bold. See Table 2 for a description of the predictor variables. Out of the 702 observations, there were no speed measurements in 7 cases, hence the sample size of 695 for model S3.

The best model for three behavioural classes, based on speed alone (model S3), classified 470 out of 695 observations correctly (7 out of the 702 observations in our dataset did not have a speed measurement) resulting in an absolute cross-validation error of 0.35. Speeds below 0.18 m s^−1^ were classified as no locomotion, speeds higher or equal to 3.4 m s^−1^ as fly and intermediate speeds as terrestrial locomotion (Figure 3A). Fly was classified incorrectly as terrestrial locomotion in 8% (1 out of 13) of the cases. Observed behaviours belonging to the terrestrial locomotion group were incorrectly classified as no locomotion in 44% of the cases and behaviours belonging to the no locomotion group were incorrectly classified as terrestrial locomotion in 24% of the cases.

### 3-class acceleration model

The best model for three behavioural classes, using speed and acceleration data (model SA3), classified 609 of the 702 observations correctly (absolute cross-validation error = 0.14). Observed behaviours belonging to the terrestrial locomotion group were incorrectly classified as no locomotion in 9% of the cases, and no locomotion observations were incorrectly classified as terrestrial locomotion in 18% of the cases. The predictors that were included in the model were the mean dynamic acceleration in the surge axis (odbaX) and maximum power spectral density (psd) of dynamic acceleration along the heave axis (dpsZ) (Figure 3B, Table 2). If odbaX was less than 0.09 g, equivalent to no dynamic acceleration in the surge axis, then behaviour was classified as no movement, if odbaX was higher and dpsZ was greater than or equal to 5.1 W Hz^−1^, then behaviour was classified as fly and if odbaX was greater than or equal to 0.09 g and dpsZ was less than 5.1 W Hz−1, then behaviour was classified as terrestrial locomotion (Figure 3B). Speed was not retained as a predictor variable.

### 8-class acceleration model

**Figure 4:**
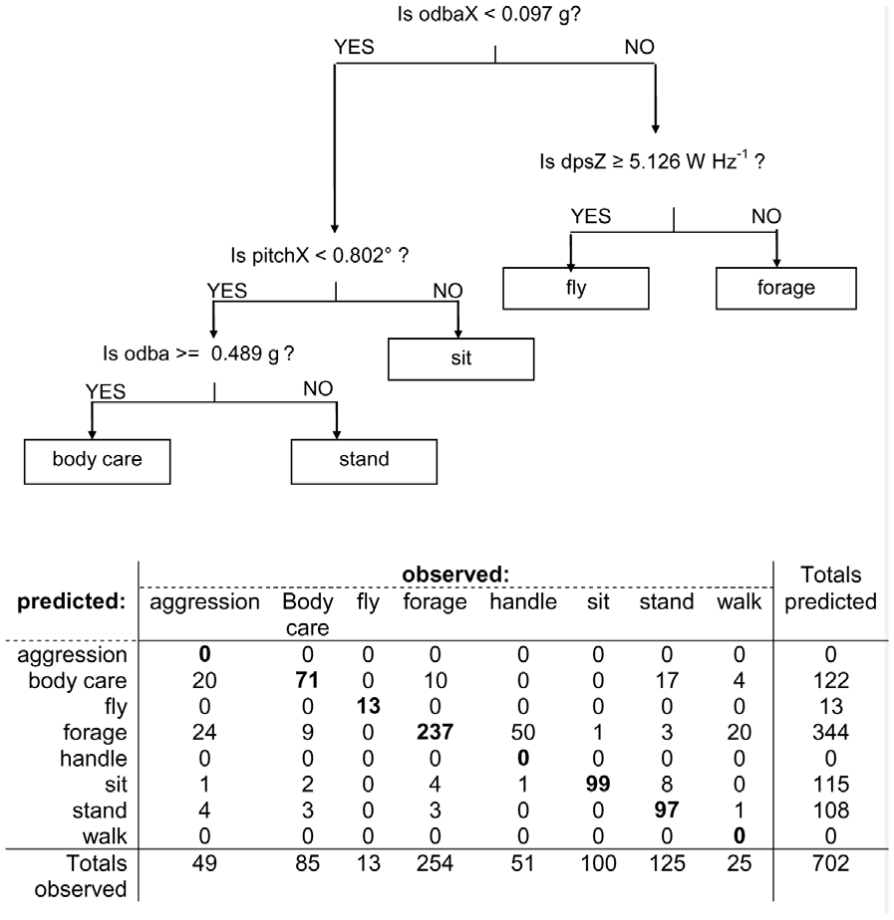
Decision tree and confusion matrix for model SA8. The number of observations correctly classified per behaviour is shown in bold. See Table 2 for a description of the predictor variables.

The best model for the eight behavioural classes, using speed and acceleration data (model SA8), classified 517 of the 702 observations correctly (absolute cross-validation error = 0.282). Only five of the eight behaviours were classified: ‘fly’, ‘forage’, ‘body care’, ‘stand’ and ‘sit’ (Figure 4). Walk was generally misclassified as forage which is not surprising as forage included, by definition, walking movement (see Table 1). Aggression was generally misclassified as body care or forage, and handle was predominantly misclassified as forage. From the 15 different explanatory variables, only four variables were retained in the classification model: odbaX and dpsZ, both also included in model SA3, as well as overall dynamic body acceleration (odba) and the pitch angle measured in the surge (pitchX). As with model SA3, odbaX can be used to distinguish between forward locomotion (fly and forage) and no locomotion (body care, stand and sit). Similarly, as in model SA3, dpsZ greater than or equal to 5.1 g^2^ Hz^−1^ could be used to distinguish fly from forage.

**Figure 5:**
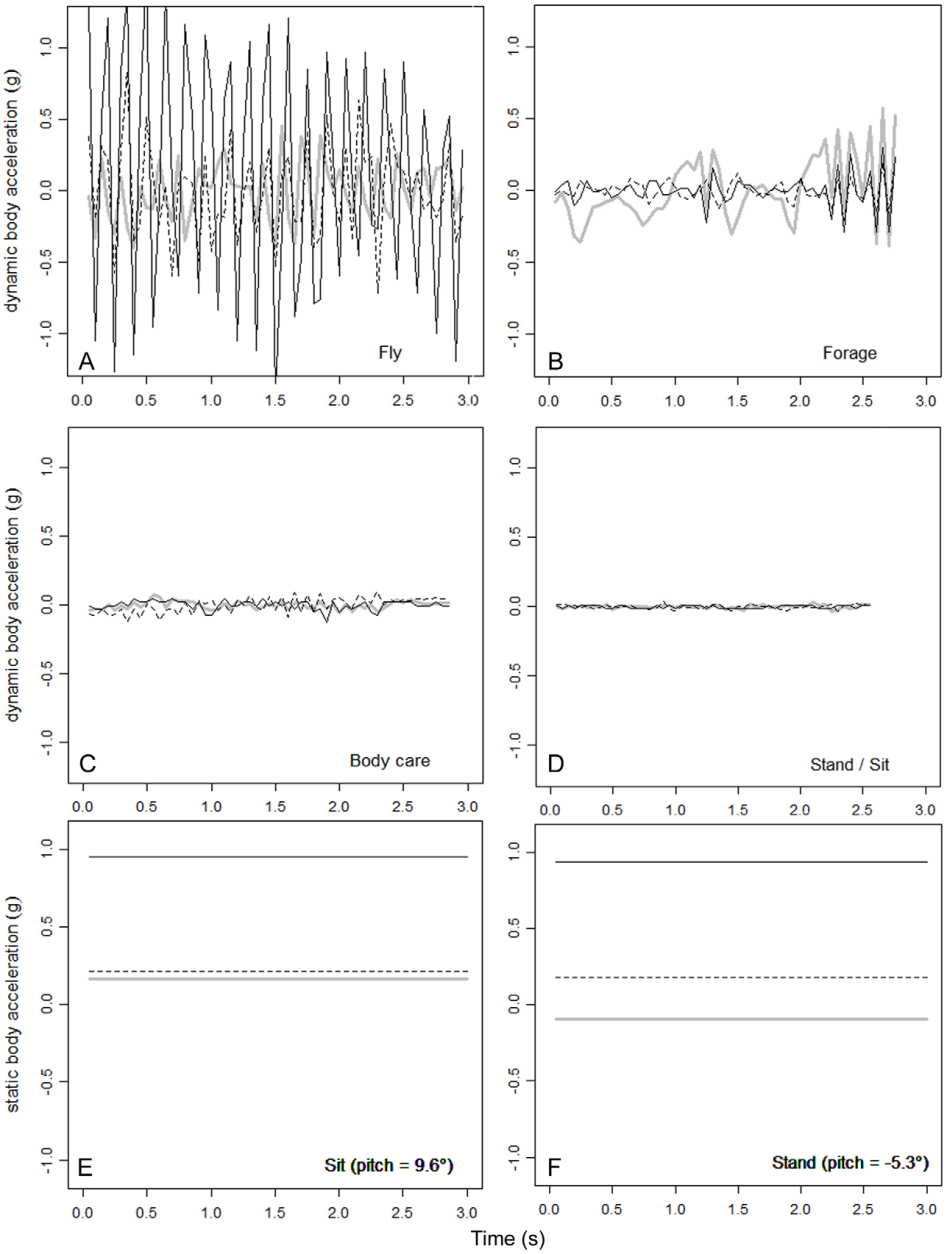
Examples of behaviours with characteristic signals from dynamic acceleration and static acceleration. Characteristic signals from dynamic acceleration (A–D) and static acceleration (E–F) are shown. In all panels, acceleration in the surge (X) axis is shown with a continuous grey line, in the sway (Y) axis with a dashed line and in the heave (Z) axis with a continuous black line. Fly and forage (A, B) are especially characterized by high-amplitudes of all dynamic acceleration components, but the frequency of the signals is higher for fly than it is for forage (especially in the Z direction, see dpsZ in Figure 4). Many of the accelerometer signals for foraging are characterized by the alternation between relatively smooth lateral movement (changes in acceleration predominantly in the surge axis) and short bursts of high frequency changes in acceleration in all three axes (e.g. catching prey, at 2.2 s panel B, see also Video S1). The changes in dynamic acceleration for body care (C) are much smaller than for fly and forage, but still considerably higher than for stand and sit (D, see also odbaX and odba in Figure 4). The static acceleration can be used to distinguish sit (E) and stand (F) due to differences in body posture (see pitchX in Figure 4).

The decision rules in this model (Figure 4) are easy to interpret within the context of the field observations and locomotion. Dynamic acceleration or deceleration in the surge axis (odbaX) describes active forward movement, and the bird is either flying or walking, depending on the amount of energy invested at the dominant periodicity in the heave axis signal (dpsZ). When there is not much movement in the heave or surge, the bird is either standing or sitting, depending on the pitch angle of the body; a zero or slightly positive angle means that the logger is horizontal and the bird is sitting and a negative angle means that the anterior of the bird is tilted upwards and the bird is standing (see Text S1 for more details). While standing, the bird may preen its feathers moving its bill and body, resulting in higher overall dynamic body acceleration (odba) than when standing still. Figure 5 shows characteristic examples of dynamic and static acceleration signals for fly, forage, body care, stand and sit behaviours, which were correctly classified by the SA8 model. See Video S1 for an example of foraging behaviour coupled with accelerometer data.

The SA8 model effectively reduced the number of behavioural classes from 8 to 5. This result and the cross validation error indicated that more detailed behavioural classes or trying to classify the 16 mutually exclusive classes of behaviour observed (column sub-behaviour in Table 1) was not feasible.

### Time budget analysis

**Figure 6:**
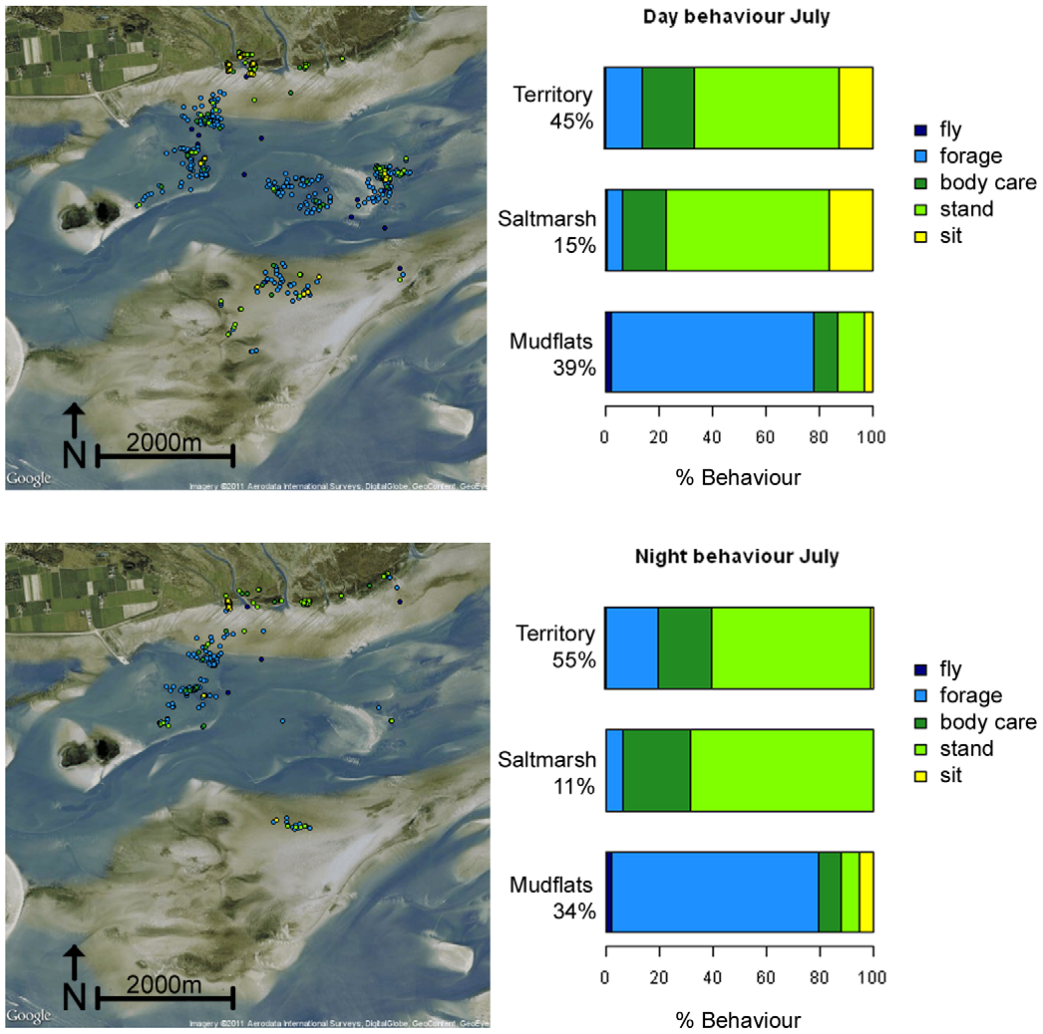
Diurnal and nocturnal time budget of one oystercatcher during July 2009, using model SA8 to classify behaviours. Diurnal (top) and nocturnal (bottom) time budgets for one oystercatcher (logger 169, Table S1) during July 2009, using model SA8 (Figure 4) to classify behaviours. The locations of each behaviour (fly, forage, body care, stand and sit) are presented on the map; the colours of the icons on the map correspond to those in the time budget bar graphs.

The SA8 model was applied to classify behaviour per accelerometer segment and then associated to the respective GPS fix (time and location). Subsequently, the time spent on five different behavioural activities (‘fly’, ‘forage’, ‘body care’, ‘stand’ and ‘sit’) in several habitats was calculated for each bird. The time budget analysis results for the bird fitted with logger 169 (a female) are presented in Figure 6 and for birds fitted with loggers 166 and 167 (both males) in Figures S1 and S2. The time budgets differed between individuals predominantly in where and when they spent their time on different activities rather than the total proportion of time spent on any one activity. All three birds spent a similar proportion of time during the day foraging as during the night (166: 39% and 45%; 167: 38% and 40%; 169: 37% and 38% respectively). Individual 169 spent most of this time foraging on the mudflats. When on the salt marsh, which functions predominantly as a roosting site outside the breeding season, the three birds spent most of their time on other activities, such as standing, sitting (barely at night) and body care (Figure 6). All three birds spent relatively little time in flight during the day and at night (<2% of total time) and spent relatively more time during the day sitting than at night. While the proportion of time spent foraging barely differed between day and night, the spatial distributions of the classified behaviours clearly differed (Figure 6).

## Discussion

### Classification models

The primary aim of this paper was to develop and assess classification models to convert sensor data into specific behaviours observed in the field. As we expected, variables derived from body acceleration are clearly better predictors of behaviour than speed alone. Thus, when tracking animals, collecting acceleration has a great added value if information about behaviour is desired. However, since many GPS tracking studies only provide information on speed and location it is useful to note that ground speed measured by the GPS can, in some cases, be used to distinguish flight from non-flight quite reliably. Yet the threshold will differ per species, flight strategy used (e.g. soaring or flapping flight [59]) and environmental conditions such as wind speed and direction. In this study, 3.4 m s^−1^ and higher is associated with flight (Figure 3a and Table S2), however this threshold is based on a very small sample of 13 observations made close to the nest. In a study on Manx Shearwaters (*Puffinus puffinus*), using a different methodology, a ground speed of 2.5 m s^−1^ was found as the optimal threshold between sitting and flying [60]. Since terrestrial locomotion in oystercatchers is quite slow (Table S2) and GPS speed is not accurate enough [26], distinguishing between terrestrial locomotion and no locomotion is more difficult.

The models we developed can be applied to automatically classify additional sensor data from the same individuals and potentially the same species. However, as with any model, if the dataset used to fit the model is very limited, for example in the number of measurements per behaviour or the environmental conditions experienced, the chance of misclassification may increase. In general, once behaviours can be reliably classified, the locomotion parameters such as flight speed, wing beat frequency, gait rates, odba can then be used for comparative analysis between species, individuals, environmental conditions or for comparison with theoretical estimates [37], [40], [61]–[63]. One aspect which deserves more attention in the future, especially when samples are large enough, is the extent to which predictor variables differ within and between individuals. If predictor variables differ significantly between individuals and enough data is available, then building and applying models per individual may result in lower classification errors than when using models calibrated on pooled data. However, if predictive variables are robust enough, they could encompass individual variability.

Unfortunately, in the current study we could not derive a reliable model that could classify the 16 sub-behaviours observed in the field, and could only classify five of the eight main behaviours. Nevertheless, classification models could potentially be improved in several ways in the future. We strongly believe that video observations would be extremely useful for classifying behaviour, developing predictor variables, and re-evaluating models [44]–[45]. As video can be observed again after the activity has taken place, synchronization between observations and measurements can be improved, observations and interpretations can be cross-validated and the importance of context (for example presence of other individuals, or past events) can also be considered when classifying behaviour. As humans we are not always conscious of all the information we are visually processing to reach a certain conclusion and yet when only using part of this information for automated classification we expect the same conclusions to emerge. By re-examining videos carefully we may be able to identify these gaps and fill them. For example, studying posture, properties of movement and the measurements simultaneously (see video S1), may provide a better understanding of how they are related and enable researcher to derive more suitable predictor variables. The predictor variables included in this study are all aggregate measures which, for example, do not parameterize dependencies within the 3 s observation period, and are hence crude in some respects. A good example is given in Figure 5B, showing alternating patterns (with regard to total energy as well as frequency) of acceleration within the 3-second observation period. Thus, predictor variables which account for dependencies within an acceleration segment may also result in model improvement.

### Quantifying behaviour in space and time

By combing information on the location of the bird and the time from the GPS, behaviour from the accelerometer and information about the environment we can calculate spatio-temporal activity budgets for comparative analysis. The strength of this approach is that once a classification model is built, it can be applied to data where additional observations (visual or video) are not available or not possible. In the current study we apply the classification model to data from three individuals for which simultaneous observations were not always available. One aspect we were interested in was a comparison of diurnal and nocturnal time budgets as oystercatchers are known to forage at night in tidal areas. In a GPS tracking study of oystercatchers in the Wadden Sea [64], the authors showed that oystercatchers travel farther at night than during the day, suggesting that they foraged extensively at night, although information on behaviour was not available. In our study, we showed that although individuals visited different locations during the day and night, all three individuals spent similar proportions of time foraging during the night as during the day (Figures 6, S1, S2). Our study also showed that the three individuals spent very little time in flight (>2%) both inside and outside of the territory, which is similar to findings from a time budget analyses based on visual observations within the territory and immediate surroundings [53], [65]. Furthermore, our study supports previous suggestions that oystercatchers forage predominantly in their territory and in the mudflats close by [53], [65]. While we cannot generalize these results on the basis of the small sample used in this case study, it shows how these methods can be used to compare time budgets within and between individuals. In the future, we will apply the classification model in the future to a longer time series and more individuals to study inter-seasonal carry-over effects of habitat selection and time-activity budgets. In this context, the type of tracking system is very relevant, the UvA-BiTS enables the retrieval of data or re-programming the sensors remotely while with most of the commercially available tracking equipment an individual must be recaptured to retrieve the data (e.g. [27], [64]).

### Methodological workflow

The methodological workflow presented here can be used for similar studies regardless of the study species or the environment in which the study is conducted (e.g. terrestrial or marine). By implementing such a workflow in a programming language with a connection to a database where the data is stored, the researcher greatly facilitates the reproducibility of results, re-analysis, model improvement, knowledge transfer and collaboration, especially for researchers first entering the field of bio-logging. To facilitate the transfer of knowledge, we have provided a modelling package (Dataset S1) which includes a database and R-scripts written to run the analysis in this study. As shown in this study, several processes may be iterative, such as the specification of model predictors or model design; each iteration may improve our understanding and interpretation of the data as well as our models and a methodological workflow can streamline this process.

### Concluding remarks

The application of accelerometers in behavioural research has greatly increased in the last few years. Similarly, new developments at the interface of ecology and computer science may greatly facilitate the analysis, visualization and exploration of such data [46], [66]. Recent studies have also shown that measures of dynamic body acceleration can be used to estimate energy expenditure in a number of species during active locomotion as well as more sedentary behaviour [3], [28], [30], [67]–[68]. Thus, the potential for using accelerometers to quantify behaviour and energy expenditure makes it a very powerful tool in ecological research. Once different characteristics of behaviour and body locomotion are quantified they can be compared between studies, individuals, species, environmental conditions, seasons or even different life history stages such as migratory compared to foraging movements. Comparative studies may also help increase our understanding of biomechanics and evolution of locomotion [37]. Perhaps most exciting is the possibility to link behaviour and energy expenditure to space use and time at the individual level to gain new insight into the ability of animals to adapt to an ever changing world. In this study we provided a blueprint for the development and application of classification models for this purpose.

## Supporting Information

Text S1 Extended methods.
(PDF)

Figure S1 **Diurnal and nocturnal time budget of one oystercatcher during July 2009, using model SA8 to classify behaviours.** Diurnal (top) and nocturnal (bottom) time budgets for one oystercatcher (logger 166, Table S1) during July 2009, using model SA8 (Figure 4) to classify behaviours. The locations of each behaviour (fly, forage, body care, stand and sit) are presented on the map; the colours of the icons on the map correspond to those in the time budget graph.
(PDF)

Figure S2 **Diurnal and nocturnal time budget of one oystercatcher during July 2009, using model SA8 to classify behaviours.** Diurnal (top) and nocturnal (bottom) time budgets for one oystercatcher (logger 167, Table S1) during July 2009, using model SA8 (Figure 4) to classify behaviours. The locations of each behaviour (fly, forage, body care, stand and sit) are presented on the map; the colours of the icons on the map correspond to those in the time budget graph.
(PDF)

Table S1 The total number of GPS fixes and accelerometer segments (3 s intervals) obtained from the date of deployment through 31 July 2009 for each of the three oystercatchers in this study. Individual ring code, logger number, sex and body mass (g) on date of deployment are also provided.
(PDF)

Table S2 List of behaviours observed in the field and the mean and standard deviation of the predictor variables per behaviour according to Table 1 as follows: Table S2-A, 3-class model (S3 and SA3) behaviours (Table 1 column 4); Table S2-B, behaviours for SA8 model (Table 1 column 1); Table S2-C, 16 sub behaviours (Table 1, column 2). The predictor variables are described in Table 2.
(PDF)

Dataset S1 **A dataset and software package.** The R-scripts and dataset for this study can be found in this self-contained archive which also includes a readme-file that explains its contents.
(ZIP)

Video S1 A short video of an oystercatcher foraging by sight (Table 1) shown simultaneously with corresponding dynamic and static acceleration in the heave axis (green), surge axis (red) and sway axis (blue) in units of *g* (1 *g* = 9.8 m/s^2^). The measurement duration is 10 s, the film is shown at a slower rate. This record was not included in this study.
(WMV)

## References

[1] Norberg RA (1977). An ecological theory on foraging time and energetics and choice of optimal food-searching method.. J Anim Ecol.

[2] Ropert-Coudert Y, Grémillet D, Kato A, Ryan PG, Naito Y (2004). A fine-scale time budget of Cape gannets provides insights into the foraging strategies of coastal seabirds.. Anim Behav.

[3] Halsey LG, White CR (2010). Measuring Energetics and Behaviour Using Accelerometry in Cane Toads *Bufo marinus*.. PLoS ONE.

[4] Aviles JM (2004). Common cranes Grus grus and habitat management in holm oak dehesas of Spain.. Biodivers Conserv.

[5] Bograd SJ, Block BA, Costa DP, Godley BJ (2010). Biologging technologies: new tools for conservation. Introduction.. Endangered Species Research.

[6] Pichegru L, Ryan P, der Lingen C, Coetzee J, Ropert-Coudert Y (2007). Foraging behaviour and energetics of Cape gannets *Morus capensis* feeding on live prey and fishery discards in the Benguela upwelling system.. Mar Ecol-Prog Ser.

[7] Gaidet N, Cappelle J, Takekawa JY, Prosser DJ, Iverson SA (2010). Potential spread of highly pathogenic avian influenza H5N1 by wildfowl: dispersal ranges and rates determined from large-scale satellite telemetry.. J Appl Ecol.

[8] Cumming GS, Hockey PAR, Bruinzeel LW, Du Piessis MA (2008). Wild bird movements and avian influenza risk mapping in Southern Africa.. Ecol Soc.

[9] McLellan BN, Shackleton DM (1988). Grizzly bears and resource-extraction industries: effects of roads on behaviour, habitat use and demography.. J Appl Ecol.

[10] Sutherland WJ, Armstrong-Brown S, Armsworth PR, Tom B, Brickland J (2006). The identification of 100 ecological questions of high policy relevance in the UK.. J Appl Ecol.

[11] Weimerskirch H, Ancel A, Caloin M, Zahariev A, Spagiari J (2003). Foraging efficiency and adjustment of energy expenditure in a pelagic seabird provisioning its chick.. J Anim Ecol.

[12] Camphuysen CJ, Shamoun-Baranes J, Bouten W, Garthe S (2012). Identifying ecologically important marine areas for seabirds using behavioural information in combination with distribution patterns.. Biol Conserv.

[13] Robinson WD, Bowlin MS, Bisson I, Shamoun-Baranes J, Thorup K (2010). Integrating concepts and technologies to advance the study of bird migration.. Front Ecol Environ.

[14] Millspaugh JJ, Marzluff JM (2001). Radio tracking and animal populations.

[15] Kuhn CE, Johnson DS, Ream RR, Gelatt TS (2009). Advances in the tracking of marine species: using GPS locations to evaluate satellite track data and a continuous-time movement model.. Mar Ecol-Prog Ser.

[16] Hart KM, Hyrenbach KD (2009). Satellite telemetry of marine megavertebrates: the coming of age of an experimental science.. Endangered Species Research.

[17] Bridge ES, Thorup K, Bowlin MS, Chilson PB, Diehl RH (2011). Technology on the Move: Recent and Forthcoming Innovations for Tracking Migratory Birds.. BioScience.

[18] Rutz C, Hays GC (2009). New frontiers in biologging science.. Biol Lett.

[19] Ropert-Coudert Y, Wilson RP (2005). Trends and perspectives in animal-attached remote sensing.. Front Ecol Environ.

[20] Cooke SJ, Hinch SG, Wikelski M, Andrews RD, Kuchel LJ (2004). Biotelemetry: a mechanistic approach to ecology.. Trends Ecol Evol.

[21] Klaassen R, Strandberg R, Hake M, Alerstam T (2008). Flexibility in daily travel routines causes regional variation in bird migration speed.. Behav Ecol Sociobiol.

[22] Shamoun-Baranes J, Baharad A, Alpert P, Berthold P, Yom-Tov Y (2003). The effect of wind, season and latitude on the migration speed of white storks *Ciconia ciconia*, along the eastern migration route.. J Avian Biol.

[23] Thorup K, Alerstam T, Hake M, Kjellen N (2006). Traveling or stopping of migrating birds in relation to wind: an illustration for the osprey.. Behav Ecol.

[24] Shamoun-Baranes J, Bouten W, Camphuysen CJ, Baaij E (2011). Riding the tide: intriguing observations of gulls resting at sea during breeding.. Ibis.

[25] Guilford T, Meade J, Willis J, Phillips RA, Boyle D (2009). Migration and stopover in a small pelagic seabird, the Manx shearwater *Puffinus puffinus*: insights from machine learning.. P Roy Soc B-Biol Sci.

[26] Witte TH, Wilson AM (2004). Accuracy of non-differential GPS for the determination of speed over ground.. J Biomech.

[27] Wilson RP, Shepard ELC, Liebsch N (2008). Prying into the intimate details of animal lives: use of a daily diary on animals.. Endangered Species Research.

[28] Green JA, Halsey LG, Wilson RP, Frappell PB (2009). Estimating energy expenditure of animals using the accelerometry technique: activity, inactivity and comparison with the heart-rate technique.. J Exp Biol.

[29] Bidder OR, Soresina M, Shepard ELC, Halsey LG, Quintana F (2012). The need for speed: testing acceleration for estimating animal travel rates in terrestrial dead-reckoning systems.. Zoology.

[30] Gleiss AC, Wilson RP, Shepard ELC (2011). Making overall dynamic body acceleration work: on the theory of acceleration as a proxy for energy expenditure.. Methods in Ecology and Evolution.

[31] Ropert-Coudert Y, Daunt F, Kato A, Ryan PG, Lewis S (2009). Underwater wingbeats extend depth and duration of plunge dives in northern *Morus bassanus*.. J Avian Biol.

[32] Gómez Laich A, Wilson RP, Quintana F, Shepard ELC (2008). Identification of imperial cormorant *Phalacrocorax atriceps* behaviour using accelerometers.. Endangered Species Research.

[33] Mitani Y, Andrews RD, Sato K, Kato A, Naito Y (2010). Three-dimensional resting behaviour of northern elephant seals: drifting like a falling leaf.. Biol Lett.

[34] Weimerskirch H, Le Corre M, Ropert-Coudert Y, Kato A, Marsac F (2005). The three-dimensional flight of red-footed boobies: adaptations to foraging in a tropical environment?. P Roy Soc B-Biol Sci.

[35] Yoda K, Kohno H, Naito Y (2004). Development of flight performance in the brown booby.. P Roy Soc B-Biol Sci.

[36] Miller PJO, Johnson MP, Tyack PL, Terray EA (2004). Swimming gaits, passive drag and buoyancy of diving sperm whales *Physeter macrocephalus*.. J Exp Biol.

[37] Gleiss AC, Jorgensen SJ, Liebsch N, Sala JE, Norman B (2011). Convergent evolution in locomotory patterns of flying and swimming animals.. Nat Commun.

[38] Okuyama J, Kawabata Y, Naito Y, Arai N, Kobayashi M (2009). Monitoring beak movements with an acceleration datalogger: a useful technique for assessing the feeding and breathing behaviors of sea turtles.. Endangered Species Research.

[39] Whitney N, Pratt HJ, Pratt T, Carrier J (2010). Identifying shark mating behaviour using three-dimensional acceleration loggers.. Endangered Species Research.

[40] Ropert-Coudert Y, Beaulieu M, Hanuise N, Kato A (2009). Diving into the world of biologging.. Endangered Species Research.

[41] Holland RA, Wikelski M, Kümmeth F, Bosque C (2009). The Secret Life of Oilbirds: New Insights into the Movement Ecology of a Unique Avian Frugivore.. PLoS ONE.

[42] Shepard ELC, Wilson RP, Quintana F, Gómez Laich A, Liebsch N (2008). Identification of animal movement patterns using tri-axial accelerometry.. Endangered Species Research.

[43] Shepard ELC, Lambertucci SA, Vallmitjana D, Wilson RP (2011). Energy Beyond Food: Foraging Theory Informs Time Spent in Thermals by a Large Soaring Bird.. PLoS ONE.

[44] Yoda K, Naito Y, Sato K, Takahashi A, Nishikawa J (2001). A new technique for monitoring the behaviour of free-ranging Adelie penguins.. J Exp Biol.

[45] Halsey LG, Portugal SJ, Smith JA, Murn CP, Wilson RP (2009). Recording raptor behavior on the wing via accelerometry.. J Field Ornithol.

[46] Sakamoto KQ, Sato K, Ishizuka M, Watanuki Y, Takahashi A (2009). Can Ethograms Be Automatically Generated Using Body Acceleration Data from Free-Ranging Birds?. PLoS ONE.

[47] Watanabe S, Izawa M, Kato A, Ropert-Coudert Y, Naito Y (2005). A new technique for monitoring the detailed behaviour of terrestrial animals: A case study with the domestic cat.. Appl Anim Behav Sci.

[48] Hagemeijer WJM, Blair MJ (1997). The EBCC Atlas of European Breeding Birds: their Distribution and Abundance.

[49] Ens BJ, Safriel UN, Harris MP (1993). Divorce in the long-lived and monogamous oystercatcher, *Haematopus ostralegus*: incompatibility or choosing the better option?. Anim Behav.

[50] Heg D, Ens BJ, Van Der Jeugd HP, Bruinzeel LW (2000). Local dominance and territorial settlement of nonbreeding oystercatchers.. Behaviour.

[51] Ens BJ, Kersten M, Brenninkmeijer A, Hulscher JB (1992). Territory quality, parental effort and reproductive success of Oystercatchers (*Haematopus ostralegus*).. J Anim Ecol.

[52] Pol Mvd, Brouwer L, Ens BJ, Oosterbeek K, Tinbergen JM (2009). Fluctuating selection and the maintenance of individual and sex-specific diet specialization in free-living oystercatchers.. Evolution.

[53] Kersten M (1996). Time and energy budgets of oystercatchers Haematopus ostralegus occupying territories of different quality.. Ardea.

[54] Breiman L, Friedman JH, Olshen RA, Stone CJ (1984). Classification and regression trees.

[55] Prasad A, Iverson L, Liaw A (2006). Newer Classification and Regression Tree Techniques: Bagging and Random Forests for Ecological Prediction.. Ecosystems.

[56] Themeau TM, Atkinson EJ (1997). An introduction to recursive partitioning using the RPART routines.. Department of Health Sciences Research, Rochester, MN: Mayo Clinic.

[57] R Development Core Team (2011). R: A language and environment for statistical computing. R Foundation for Statistical Computing, Vienna, Austria. ISBN 3-900051-07-0 (R website.. http://www.R-project.org/.

[58] Hastie T, Tibshirani R, Friedman J (2009). The Elements of Statistical Learning: Data Mining, Inference and Prediction.

[59] Shamoun-Baranes J, van Loon E (2006). Energetic influence on gull flight strategy selection.. J Exp Biol.

[60] Guilford TC, Meade J, Freeman R, Biro D, Evans T (2008). GPS tracking of the foraging movements of Manx Shearwaters *Puffinus puffinus* breeding on Skomer Island, Wales.. Ibis.

[61] Bruderer B, Boldt A (2001). Flight characteristics of birds: I. radar measurements of speeds.. Ibis.

[62] Bruderer B, Peter D, Boldt A, Liechti F (2010). Wing-beat characteristics of birds recorded with tracking radar and cine camera.. Ibis.

[63] Zijlstra W, Hof AL (2003). Assessment of spatio-temporal gait parameters from trunk accelerations during human walking.. J Biomech.

[64] Schwemmer P, Garthe S (2011). Spatial and temporal patterns of habitat use by Eurasian oystercatchers (*Haematopus ostralegus*) in the eastern Wadden Sea revealed using GPS data loggers.. Mar Biol.

[65] Kersten M, Visser W (1996). Food intake by Oystercatchers *Haematopus ostralegus* by day and by night measured with an electronic nest balance.. Ardea.

[66] Shamoun-Baranes J, van Loon EE, Purves RS, Speckmann B, Weiskopf D (2012). Analysis and visualization of animal movement.. Biol Lett.

[67] Fossette S, Schofield G, Lilley MKS, Gleiss AC, Hays GC (2012). Acceleration data reveal the energy management strategy of a marine ectotherm during reproduction.. Funct Ecol.

[68] Wilson RP, White CR, Quintana F, Halsey LG, Liebsch N (2006). Moving towards acceleration for estimates of activity-specific metabolic rate in free-living animals: the case of the cormorant.. J Anim Ecol.

