# Supplementary material for: From Sensor Data to Animal Behaviour: An Oystercatcher Example
Source: PLoS One. 2012 May 31;7(5):e37997. doi: 10.1371/journal.pone.0037997 (PMC3365100; doi:10.1371/journal.pone.0037997)
Supplement: Text S1 — Extended methods. (PDF) [file pone.0037997.s001.pdf]

Text S1. Extended Methods

Data collection

The tracking devices communicate with a base station via a Zigbee wireless connection, transmitting data and receiving new measurement programs. Communication between the base station and tracking devices was facilitated by three antennas, one on each of two observation towers that were standing within 50 m from the nests, and one above the base station situated within 1 km from the observation towers (Figure 1).

Data Processing

In order to merge the GPS and accelerometer data with visual observations we had to account for a potential time lag between the initiation of a new behaviour measured by the tracking device and recording the behaviour on the handheld computer. This time lag was calibrated during two days when the loggers were set to record continuous tri-axial acceleration for 30 minutes. With this data it was possible to distinguish very different behaviours, such as the transition from walking to flying, and thus, to measure the time lag between the activity measured by the tracking device and the activity recorded on the handheld computer (mean ± SD, 5 ± 2 s, n = 62, range 0-10 s). This means that visual observations recorded on the handheld computer at time *t* potentially began at time *t* - 10 s as recorded by the sensor (GPS and accelerometer). Since the tracking device and handheld computer clocks were synchronized, the two datasets could be temporally aligned. Each sensor measurement was then labelled with a behavioural class at *t*-10 s that corresponded to the observed behaviour recorded at *t* on the handheld computer. As a result of the recording delay, not all observed behaviours could be linked unambiguously to sensor data. The observations lasting less than 10 seconds were filtered out of the data set that was used in further analysis

Modelling

In order to derive predictor variables from the accelerometer, the accelerometer data were first converted into units of *g* (1 *g* = 9.8 m s^{-2}), acceleration due to gravity, by dividing the measurements by the average calibration value for these devices (1350 mV). The measured acceleration is the result of both a static and a dynamic component [1]. The static component is a measure of the incline of the accelerometer with respect to the earth's gravitational field. In this study, static acceleration in each axis was calculated as the average acceleration per 3 s acceleration segment. Static acceleration enables a calculation of the body orientation in the pitch and roll (see [1] for a detailed description). In this study a pitch of 0° means that the logger

is in a horizontal position. When a logger is vertical and the antenna points downwards (i.e. the anterior of the bird points upwards), the pitch angle is -90° (-1 g), when the antenna points upwards (the posterior of the bird points upwards) the pitch angle is 90° (1 g). Dynamic acceleration was then calculated for each axis independently by subtracting the static acceleration (the mean acceleration for 3 s) from each acceleration measurement (60 per 3 s segment). These values were then used to calculate the maximum dynamic body acceleration, the mean dynamic body acceleration (odba) per axis and the odba (Table 2). The power spectral density was calculated by the squared magnitude of the fast Fourier transform acceleration in each axis. The power spectral density measures the frequency content of a signal and can be used to detect periodicities in that signal (strong periodicities appear as peaks in the graph of power spectral density versus frequency). In this study we identified the maximum power spectral density (called 'dominant power spectrum') to express the relative amount of kinetic energy that was spent at the dominant periodicity as well as the frequency of the dominant periodicity ('frequency at the dominant power spectrum').

Deleted: *Model application*¶
Since the tracking devices were programmed to log on a regular time interval of 10 minutes for the period July 1 to 21 and 30 minutes for the period July 21-31. The percentage of time devoted to each of the classified behaviours was calculated by dividing the number of observations per behaviour by the total number of observations during the day or during the night. We associated each GPS fix to one of the following habitats: territory, mudflats and salt marsh using the geographic database of global administrative areas (GADM, http://www.gadm.org). A bird was considered to be in its territory when it was within 150 m from its nest. This distance was chosen after visually inspecting the locations of each individual in relation to their nest. Day was defined as the hours between sunrise and sunset at Schiermonnikoog (53.47 N, 6.23 E).¶

References

1. Shepard ELC, Wilson RP, Quintana F, Gómez Laich A, Liebsch N, et al. (2008) Identification of animal movement patterns using tri-axial accelerometry. Endangered Species Research 10: 47-60.
