# Supplementary material for: From Sensor Data to Animal Behaviour: An Oystercatcher Example
Source: PLoS One. 2012 May 31;7(5):e37997. doi: 10.1371/journal.pone.0037997 (PMC3365100; doi:10.1371/journal.pone.0037997)

Figure S2. . **Diurnal and nocturnal time budget of one oystercatcher during July 2009, using model SA8 to classify behaviours.** Diurnal (top) and nocturnal (bottom) time budgets for one oystercatcher (logger 167, Table S1) during July 2009, using model SA8 (Figure 4) to classify behaviours. The locations of each behaviour (fly, forage, body care, stand and sit) are presented on the map; the colours of the icons on the map correspond to those in the time budget graph.

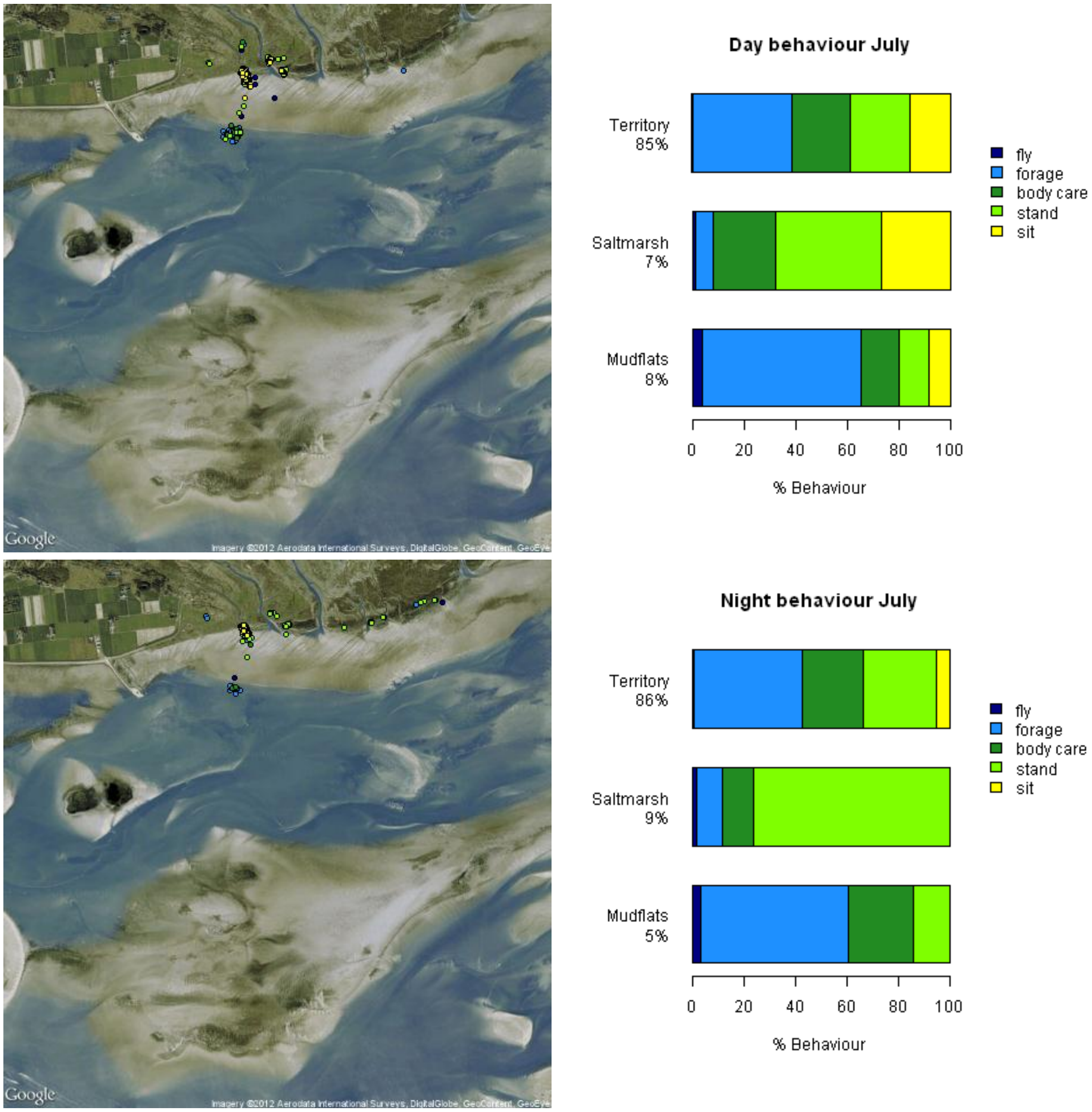

Supplement: Figure S2 — Diurnal and nocturnal time budget of one oystercatcher during July 2009, using model SA8 to classify behaviours. Diurnal (top) and nocturnal (bottom) time budgets for one oystercatcher (logger 167, Table S1) during July 2009, using model SA8 (Figure 4) to classify behaviours. The locations of each behaviour (fly, forage, body care, stand and sit) are presented on the map; the colours of the icons on the map correspond to those in the time budget graph. (PDF) [file pone.0037997.s003.pdf]
