# Supplementary material for: From Sensor Data to Animal Behaviour: An Oystercatcher Example
Source: PLoS One. 2012 May 31;7(5):e37997. doi: 10.1371/journal.pone.0037997 (PMC3365100; doi:10.1371/journal.pone.0037997)
Supplement: Table S1 — The total number of GPS fixes and accelerometer segments (3 s intervals) obtained from the date of deployment through 31 July 2009 for each of the three oystercatchers in this study. Individual ring code, logger number, sex and body mass (g) on date of deployment are also provided. (PDF) [file pone.0037997.s004.pdf]

Supporting Information

Table S1. The total number of GPS fixes and accelerometer segments (3 s intervals) obtained from the date of deployment through 31 July 2009 for each of the three oystercatchers in this study. Individual ring code, logger number, sex and body mass (g) on date of deployment are also provided.

Ring code	Logger #	Sex	Body mass (g)	Date of deployment	GPS	Accelero-meter
WR101RW3	166	Male	483	30 June	3933	2496
GW111W3	167	Male	577	29 June	3652	2413
GB021B3	169	Female	502	29 June	3835	2418
