# Supplementary material for: From Sensor Data to Animal Behaviour: An Oystercatcher Example
Source: PLoS One. 2012 May 31;7(5):e37997. doi: 10.1371/journal.pone.0037997 (PMC3365100; doi:10.1371/journal.pone.0037997)
Supplement: Table S2 — List of behaviours observed in the field and the mean and standard deviation of the predictor variables per behaviour according to Table 1 as follows: Table S2-A, 3-class model (S3 and SA3) behaviours (Table 1 column 4); Table S2-B, behaviours for SA8 model (Table 1 column 1); Table S2-C, 16 sub behaviours (Table 1, column 2). The predictor variables are described in Table 2. (PDF) [file pone.0037997.s005.pdf]

Supporting Information

Table S2. List of behaviours observed in the field and the mean and standard deviation of the predictor variables per behaviour according to Table 2 as follows: Table S2-A, 3-class model (S3 and SA3) behaviours (Table 2 column 4); Table S2-B, behaviours for SA8 model (Table 2 column 1); Table S2-C, 16 sub behaviours (Table 2, column 2). The predictor variables are described in Table 2.

Table S2-A. Predictor variables for 3-class model (S3 and SA3) behaviours (Table 1 column 4)

		Behavioural categories		
**variable**	**statistic**	**fly**	**no locomotion**	**terrestrial locomotion**
**nr. obs**	-	13	376	313
**speed***	avg	10.929	0.150	0.262
	sd	4.547	0.139	0.198
**pitchZ**	avg	10.005	19.464	20.111
	sd	10.220	6.289	4.965
**pitchX**	avg	4.739	-2.996	5.646
	sd	2.095	12.617	10.947
**rollY**	avg	2.576	-1.879	0.020
	sd	11.961	10.506	9.933
**mdbaX**	avg	0.628	0.218	0.748
	sd	0.194	0.420	0.518
**mdbaY**	avg	0.983	0.136	0.355
	sd	0.295	0.207	0.221
**mdbaZ**	avg	1.370	0.127	0.366
	sd	0.206	0.205	0.228
**odbaX**	avg	0.262	0.055	0.191
	sd	0.091	0.087	0.092

**odbaY**	avg	0.311	0.034	0.092
	sd	0.082	0.043	0.043
**odbaZ**	avg	0.820	0.033	0.103
	sd	0.116	0.054	0.072
**odba**	avg	1.393	0.122	0.386
	sd	0.223	0.174	0.187
**dpsX**	avg	1.283	0.120	0.542
	sd	0.917	0.329	0.572
**fdpsX**	avg	7.279	2.706	3.525
	sd	0.791	2.977	3.068
**dpsY**	avg	1.080	0.031	0.097
	sd	0.661	0.128	0.108
**fdpsY**	avg	6.905	3.810	4.739
	sd	1.633	3.297	3.431
**dpsZ**	avg	14.256	0.044	0.186
	sd	8.236	0.271	0.397
**fdpsZ**	avg	7.230	5.661	6.474
	sd	0.723	3.020	2.712

* for speed, 7 values are missing: 5 for the category no locomotion and 2 for terrestrial locomotion

Table S2-B. Predictor variables for behaviours for SA8 model (Table 1 column 1)

		Behavioural categories*							
**variable**	**statistic**	**aggression**	**body care**	**fly**	**forage**	**handle**	**sit**	**stand**	**walk**
**nr. obs**	-	49	85	13	254	51	100	125	25
**speed****	avg	0.246	0.171	10.929	0.248	0.228	0.134	0.115	0.361
	sd	0.188	0.168	4.547	0.186	0.164	0.091	0.129	0.267
**pitchZ**	avg	18.727	26.391	10.005	20.548	21.920	15.214	17.303	17.229
	sd	7.016	5.616	10.220	4.857	5.943	2.773	3.642	3.389
**pitchX**	avg	-7.531	-14.765	4.739	8.219	12.460	7.445	-8.381	-6.209
	sd	9.306	10.013	2.095	9.627	10.255	4.054	6.287	6.280
**rollY**	avg	-0.020	5.247	2.576	0.783	-0.913	-4.741	-5.261	-6.636
	sd	8.439	10.787	11.961	10.334	9.572	8.922	9.430	4.327
**mdbaX**	avg	0.380	0.223	0.628	0.815	1.024	0.025	0.055	0.395
	sd	0.442	0.258	0.194	0.522	0.616	0.040	0.087	0.232
**mdbaY**	avg	0.305	0.220	0.983	0.352	0.378	0.027	0.076	0.319
	sd	0.348	0.233	0.295	0.210	0.169	0.041	0.151	0.219
**mdbaZ**	avg	0.424	0.154	1.370	0.358	0.438	0.021	0.045	0.298
	sd	0.392	0.175	0.206	0.206	0.185	0.030	0.088	0.188
**odbaX**	avg	0.109	0.063	0.262	0.200	0.237	0.008	0.018	0.145
	sd	0.090	0.062	0.091	0.092	0.083	0.014	0.030	0.059
**odbaY**	avg	0.081	0.056	0.311	0.092	0.100	0.008	0.014	0.079
	sd	0.084	0.043	0.082	0.037	0.034	0.009	0.011	0.043
**odbaZ**	avg	0.112	0.041	0.820	0.099	0.117	0.007	0.013	0.095
	sd	0.145	0.038	0.116	0.056	0.046	0.006	0.017	0.087
**odba**	avg	0.302	0.160	1.393	0.391	0.454	0.022	0.045	0.319
	sd	0.306	0.135	0.223	0.172	0.143	0.028	0.054	0.177
**dpsX**	avg	0.238	0.112	1.283	0.591	0.662	0.003	0.016	0.293

	sd	0.361	0.292	0.917	0.606	0.546	0.019	0.091	0.196
**fdpsX**	avg	3.256	2.581	7.279	3.472	5.165	1.973	2.546	3.247
	sd	2.667	2.903	0.791	3.208	3.374	2.565	2.786	2.010
**dpsY**	avg	0.129	0.063	1.080	0.090	0.094	0.002	0.004	0.083
	sd	0.258	0.210	0.661	0.082	0.083	0.007	0.014	0.108
**fdpsY**	avg	3.267	4.942	6.905	4.752	5.757	2.621	3.529	5.272
	sd	3.125	3.761	1.633	3.441	3.214	2.327	3.234	3.351
**dpsZ**	avg	0.316	0.029	14.256	0.161	0.179	0.001	0.006	0.236
	sd	0.949	0.069	8.236	0.264	0.177	0.003	0.039	0.671
**fdpsZ**	avg	5.691	5.000	7.230	6.621	7.019	4.993	6.108	5.931
	sd	2.857	3.072	0.723	2.796	2.619	2.893	2.954	1.714

* order of the behavioural categories is the same as in Table 1

** for speed, 7 values are missing: 2 for the category forage, 2 for sit and 3 for stand

Table S2-C: Predictor variables for 16 sub behaviours (Table 1, column 2).

		Behavioural categories*															
**variable**	**statistic**	**bobbing**	**chasing**	**stand sol. piping**	**piping cer.**	**walk sol. piping**	**preen**	**wash**	**normal flight**	**by sight**	**by touch**	**handling at surface**	**handling in situ**	**walking with prey**	**sit**	**stand**	**walk**
**nr. obs**	-	4	3	18	12	12	82	3	13	249	5	15	29	7	100	125	25
**speed****	avg	0.15	0.30	0.18	0.19	0.43	0.17	0.13	10.93	0.25	0.23	0.27	0.20	0.23	0.13	0.11	0.36
	sd	0.12	0.22	0.14	0.14	0.20	0.17	0.11	4.55	0.19	0.10	0.19	0.13	0.22	0.09	0.13	0.27
**pitchZ**	avg	20.39	26.81	18.66	18.08	16.90	26.35	27.39	10.01	20.47	24.54	20.64	22.88	20.68	15.21	17.30	17.23
	sd	6.53	4.40	8.27	7.35	4.13	5.70	2.40	10.22	4.83	4.87	5.00	6.80	3.12	2.77	3.64	3.39
**pitchX**	avg	-15.67	-13.32	-5.53	-13.18	-0.72	-15.32	0.35	4.74	8.03	17.78	8.26	16.21	5.93	7.45	-8.38	-6.21
	sd	10.85	9.28	7.56	8.72	6.36	9.74	3.77	2.10	9.53	10.52	8.20	10.04	9.33	4.05	6.29	6.28
**rollY**	avg	-8.21	-0.05	4.70	-5.65	1.26	5.14	8.13	2.58	0.55	12.12	0.46	-2.75	3.73	-4.74	-5.26	-6.64
	sd	1.51	7.79	9.93	1.73	6.82	10.94	4.97	11.96	10.30	3.01	9.76	8.62	12.17	8.92	9.43	4.33
**mdbaX**	avg	0.12	0.92	0.28	0.39	0.47	0.18	1.32	0.63	0.82	0.54	1.02	1.13	0.61	0.03	0.05	0.40
	sd	0.09	1.21	0.33	0.32	0.42	0.15	0.27	0.19	0.52	0.22	0.64	0.63	0.30	0.04	0.09	0.23
**mdbaY**	avg	0.06	0.57	0.25	0.28	0.43	0.19	1.03	0.98	0.35	0.24	0.39	0.34	0.51	0.03	0.08	0.32
	sd	0.05	0.73	0.41	0.24	0.24	0.13	0.75	0.30	0.21	0.07	0.22	0.13	0.16	0.04	0.15	0.22
**mdbaZ**	avg	0.11	0.82	0.42	0.38	0.47	0.13	0.82	1.37	0.36	0.31	0.42	0.44	0.48	0.02	0.04	0.30
	sd	0.11	1.11	0.42	0.17	0.24	0.11	0.30	0.21	0.21	0.07	0.19	0.18	0.23	0.03	0.09	0.19
**odbaX**	avg	0.05	0.21	0.08	0.11	0.15	0.05	0.32	0.26	0.20	0.20	0.24	0.24	0.22	0.01	0.02	0.15
	sd	0.05	0.25	0.08	0.06	0.04	0.04	0.04	0.09	0.09	0.08	0.11	0.07	0.06	0.01	0.03	0.06
**odbaY**	avg	0.02	0.15	0.06	0.08	0.11	0.05	0.21	0.31	0.09	0.09	0.08	0.10	0.13	0.01	0.01	0.08
	sd	0.02	0.19	0.09	0.07	0.06	0.03	0.10	0.08	0.04	0.03	0.03	0.04	0.02	0.01	0.01	0.04
**odbaZ**	avg	0.02	0.30	0.09	0.09	0.14	0.04	0.20	0.82	0.10	0.11	0.10	0.12	0.15	0.01	0.01	0.10
	sd	0.02	0.43	0.14	0.06	0.09	0.02	0.05	0.12	0.06	0.06	0.05	0.04	0.03	0.01	0.02	0.09
**odba**	avg	0.10	0.66	0.24	0.28	0.41	0.14	0.73	1.39	0.39	0.41	0.43	0.46	0.49	0.02	0.04	0.32
	sd	0.08	0.87	0.31	0.18	0.16	0.08	0.18	0.22	0.17	0.16	0.18	0.14	0.06	0.03	0.05	0.18

**dpsX**	avg	0.07	0.73	0.15	0.15	0.39	0.06	1.44	1.28	0.59	0.41	0.73	0.66	0.52	0.00	0.02	0.29
	sd	0.09	1.13	0.30	0.13	0.24	0.13	0.45	0.92	0.61	0.20	0.73	0.49	0.30	0.02	0.09	0.20
**fdpsX**	avg	1.47	7.21	2.85	2.60	4.12	2.63	1.12	7.28	3.43	5.62	4.47	5.46	5.42	1.97	2.55	3.25
	sd	0.76	3.63	3.00	2.16	1.72	2.94	0.19	0.79	3.19	3.67	3.43	3.53	2.71	2.56	2.79	2.01
**dpsY**	avg	0.01	0.27	0.11	0.08	0.21	0.04	0.82	1.08	0.09	0.11	0.08	0.09	0.16	0.00	0.00	0.08
	sd	0.01	0.43	0.31	0.15	0.25	0.05	0.93	0.66	0.08	0.07	0.06	0.09	0.08	0.01	0.01	0.11
**fdpsY**	avg	1.03	4.04	3.91	3.13	3.00	5.10	0.67	6.91	4.75	4.90	4.35	5.85	8.40	2.62	3.53	5.27
	sd	0.48	4.89	3.48	3.17	2.59	3.74	0.01	1.63	3.45	3.50	3.19	3.21	1.01	2.33	3.23	3.35
**dpsZ**	avg	0.01	1.46	0.33	0.12	0.30	0.02	0.35	14.26	0.16	0.13	0.17	0.15	0.30	0.00	0.01	0.24
	sd	0.01	2.48	1.17	0.17	0.50	0.03	0.04	8.24	0.27	0.10	0.25	0.13	0.13	0.00	0.04	0.67
**fdpsZ**	avg	2.00	3.38	6.86	6.35	5.10	5.01	4.81	7.23	6.60	7.58	7.06	7.01	6.97	4.99	6.11	5.93
	sd	1.58	2.45	2.60	2.95	2.29	3.12	1.41	0.72	2.81	1.62	2.74	2.82	1.57	2.89	2.95	1.71

* order of the behavioural categories is the same as in Table 1

** for speed, 7 values are missing: 2 for the category forage by sight, 2 for sit and 3 for stand
